# Evaluation of a community-based mobile video breastfeeding intervention in Khayelitsha, South Africa: The Philani MOVIE cluster-randomized controlled trial

**DOI:** 10.1371/journal.pmed.1003744

**Published:** 2021-09-28

**Authors:** Maya Adam, Jamie Johnston, Nophiwe Job, Mithilesh Dronavalli, Ingrid Le Roux, Nokwanele Mbewu, Neliswa Mkunqwana, Mark Tomlinson, Shannon A. McMahon, Amnesty E. LeFevre, Alain Vandormael, Kira-Leigh Kuhnert, Pooja Suri, Jennifer Gates, Bongekile Mabaso, Aarti Porwal, Charles Prober, Till Bärnighausen

**Affiliations:** 1 Department of Pediatrics, Stanford University School of Medicine, Stanford, California, United States of America; 2 Heidelberg University Institute of Global Health, Faculty of Medicine and University Hospital, Heidelberg University, Heidelberg, Germany; 3 Stanford Center for Health Education, Stanford, California, United States of America; 4 Digital Medic, Stanford Center for Health Education, Cape Town, South Africa; 5 University of New South Wales, Sydney, Australia; 6 The Philani Maternal Child Health and Nutrition Trust, Khayelitsha, South Africa; 7 Institute for Life Course Health Research, Department of Global Health, Stellenbosch University, Stellenbosch, South Africa; 8 School of Nursing and Midwifery, Queens University, Belfast, United Kingdom; 9 Johns Hopkins Bloomberg School of Public Health, Baltimore, Maryland, United States of America; 10 University of Cape Town, School of Public Health and Family Medicine, Cape Town, South Africa; 11 Berkeley School of Public Health, Berkeley, California, United States of America; 12 Icahn School of Medicine at Mount Sinai, New York, New York, United States of America; 13 School of Management Studies, University of Cape Town, Cape Town, South Africa; 14 Harvard Center for Population and Development Studies, Cambridge, Massachusetts, United States of America; 15 Wellcome Trust’s Africa Health Research Institute (AHRI), KwaZulu-Natal, South Africa; Instituto de Salud Global de Barcelona, SPAIN

## Abstract

**Background:**

In South Africa, breastfeeding promotion is a national health priority. Regular perinatal home visits by community health workers (CHWs) have helped promote exclusive breastfeeding (EBF) in underresourced settings. Innovative, digital approaches including mobile video content have also shown promise, especially as access to mobile technology increases among CHWs. We measured the effects of an animated, mobile video series, the Philani MObile Video Intervention for Exclusive breastfeeding (MOVIE), delivered by a cadre of CHWs (“mentor mothers”).

**Methods and findings:**

We conducted a stratified, cluster-randomized controlled trial from November 2018 to March 2020 in Khayelitsha, South Africa. The trial was conducted in collaboration with the Philani Maternal Child Health and Nutrition Trust, a nongovernmental community health organization. We quantified the effect of the MOVIE intervention on EBF at 1 and 5 months (primary outcomes), and on other infant feeding practices and maternal knowledge (secondary outcomes). We randomized 1,502 pregnant women in 84 clusters 1:1 to 2 study arms. Participants’ median age was 26 years, 36.9% had completed secondary school, and 18.3% were employed. Mentor mothers in the video intervention arm provided standard-of-care counseling plus the MOVIE intervention; mentor mothers in the control arm provided standard of care only. Within the causal impact evaluation, we nested a mixed-methods performance evaluation measuring mentor mothers’ time use and eliciting their subjective experiences through in-depth interviews.

At both points of follow-up, we observed no statistically significant differences between the video intervention and the control arm with regard to EBF rates and other infant feeding practices [EBF in the last 24 hours at 1 month: RR 0.93 (95% CI 0.86 to 1.01, *P* = 0.091); EBF in the last 24 hours at 5 months: RR 0.90 (95% CI 0.77 to 1.04, *P* = 0.152)]. We observed a small, but significant improvement in maternal knowledge at the 1-month follow-up, but not at the 5-month follow-up. The interpretation of the results from this causal impact evaluation changes when we consider the results of the nested mixed-methods performance evaluation. The mean time spent per home visit was similar across study arms, but the intervention group spent approximately 40% of their visit time viewing videos. The absence of difference in effects on primary and secondary endpoints implies that, for the same time investment, the video intervention was as effective as face-to-face counseling with a mentor mother. The videos were also highly valued by mentor mothers and participants. Study limitations include a high loss to follow-up at 5 months after premature termination of the trial due to the COVID-19 pandemic and changes in mentor mother service demarcations.

**Conclusions:**

This trial measured the effect of a video-based, mobile health (mHealth) intervention, delivered by CHWs during home visits in an underresourced setting. The videos replaced about two-fifths of CHWs’ direct engagement time with participants in the intervention arm. The similar outcomes in the 2 study arms thus suggest that the videos were as effective as face-to-face counselling, when CHWs used them to replace a portion of that counselling. Where CHWs are scarce, mHealth video interventions could be a feasible and practical solution, supporting the delivery and scaling of community health promotion services.

**Trial registration:**

The study and its outcomes were registered at clinicaltrials.gov (#NCT03688217) on September 27, 2018.

## Abstract 2

Maya Adam and colleagues study a video intervention, delivered by community health workers, to promote breastfeeding in South Africa.

## Author summary

**Why was this study done?:**

- Perinatal home visits from trained community health workers (CHWs) have shown promise for increasing the prevalence of exclusive breastfeeding, a national health priority in South Africa.
- Video-based, mobile health interventions, incorporating narratives and entertainment–education (E–E), have demonstrated potential for engaging community members and improving health knowledge.
- Few studies have measured the effect of integrating health promotion videos into CHW workflows in underresourced settings.

**What did the researchers do and find?:**

- We developed a mobile, video breastfeeding intervention to be delivered by CHWs (“mentor mothers”) employed by an established community health program during their home visits with 1,502 pregnant participants.
- We randomized mentor mothers 1:1 to intervention and control arm. All participants served by the same mentor mother either received or did not receive the intervention.
- In addition to the causal impact evaluation using a randomized controlled trial, we performed a mixed-methods performance evaluation measuring mentor mothers’ time use and gaining insights into mentor mothers’ subjective experiences with the intervention through in-depth interviews.
- The randomized controlled trial showed no difference in effects between the intervention and control arms.
- The performance evaluation results showed that mentor mothers in the intervention arm spent approximately 40% of their visit time viewing videos with their participants.

**What do these findings mean?:**

- Instead of serving as a complement to the standard of care, mentor mothers in the video intervention group used it to replace two-fifths of their face-to-face counseling time with participants.
- The absence of difference between infant feeding outcomes in the 2 study arms implies that the video intervention was as effective as face-to-face counseling, when the CHWs used it to replace a portion of that counselling.
- Used in addition to face-to-face engagement, video interventions could boost the health promotion efforts of CHWs, and, where CHWs are not available or extremely scarce, mobile video health interventions could become an increasingly feasible and practical solution for the delivery and scaling of community health promotion services.
- Future research should explore alternative delivery channels for mobile video E–E health interventions and measure, across channels, the potential for these interventions to (a) increase access to health promotion in underresourced communities; (b) support existing CHW programs; and (c) improve health behaviors and outcomes.

## Background

The health benefits of exclusive breastfeeding (EBF) have been extensively documented [1]. Particularly in low- and middle-income countries (LMICs), exclusively breastfed infants are more likely to survive and thrive because EBF reduces their risk of gastrointestinal infections, excessive weight gain in childhood, and diabetes [1]. For mothers, breastfeeding also lowers the risk of breast and ovarian cancer, type 2 diabetes, and postpartum depression [2]. Despite the documented benefits, only 37% of infants in LMICs receive the globally recommended 6 months of EBF [1].

The rates of EBF in South Africa are among the lowest in the world [1]. Nationwide estimates range from 8% [1,3] to 32% [4]. Unsafe formula feeding and early introduction of solid foods into the child’s diet result in poor health outcomes [5] because pathogens entering the gut can cause life-threatening diarrheal disease [6]. Introducing solid foods before age 6 months can also result in nutrient deficiencies, when nutrient-rich breastmilk is displaced from the diet [7]. Reliable infant feeding data from South Africa are limited, complicating the evaluation and design of effective interventions designed to promote EBF [5]. Maternal educational interventions, including those delivered in the home, have shown promise in increasing rates of EBF and decreasing infant mortality [8–10].

In South Africa, community-based organizations employing community health workers (CHWs) have demonstrated efficacy in raising EBF prevalence within the communities they serve. The Philani Maternal Child Health and Nutrition Trust (Philani) [11,12] employs CHWs called “mentor mothers” who counsel families within their neighborhoods. Philani serves families living in Khayelitsha, a high-adversity, peri-urban settlement, with a high prevalence of HIV, poverty, and unemployment. [13] Prior research indicates that mothers receiving counseling from Philani’s mentor mothers were more likely to exclusively breastfeed than unenrolled mothers living in similar geographic regions [14]. Additionally, a pilot intervention, in 2015, demonstrated the feasibility and acceptability of equipping Philani mentor mothers with tablets loaded with health education videos [15]. Other research, conducted in South Africa, supports the potential benefit of equipping CHWs with health education videos, especially when the videos are created in collaboration with the organizations deploying the interventions [16].

Engaging target audiences through narrative approaches has proven effective in promoting healthier behaviors [17,18]. Incorporating elements of entertainment–education (E–E) augments health message delivery. Such elements include the use of narratives, visually compelling content, and persuasive messaging. In many populations, including underresourced communities, evidence-based E–E interventions may effectively change beliefs, attitudes, and behaviors [17,19–21].

Mobile technology is also increasingly incorporated into scalable health message delivery strategies [22–24]. Video content, optimized for mobile devices, is playing an increasingly important role in health education in LMICs [24–27]. The rapid adoption of mobile technology in countries like South Africa [28] has placed them front and center within mobile health (mHealth) innovations [29–31]. Free, text-based health messaging services have been successfully used to boost nationwide maternal–child health initiatives [32,33].

Recent systematic reviews of mHealth interventions have underscored the need for stronger experimental designs, preceded by feasibility studies, collaborative content development (including government partners), and integration of mHealth initiatives into existing health services [24]. Attempting to respond to these needs, we launched the Philani MOVIE, MObile Video Intervention for Exclusive breastfeeding (MOVIE) study using a large, cluster-randomized controlled design. This followed a feasibility study [15], which involved the same population of mentor mothers. Together with mothers they counsel, each mentor mother organically defined a cluster that was randomized within this pragmatic trial [34]. Further aligning with calls from the literature, the intervention tested in this trial, was developed in collaboration with local government health partners and delivered via integration within the Philani Mentor Mother Outreach Program [11]. In this study, our primary objective was to establish the effectiveness of the Philani MOVIE intervention for increasing EBF, improving other infant feeding practices, and increasing maternal knowledge among the study participants. We also aimed to characterize, using a nested, qualitative performance evaluation, the acceptability and desirability of the intervention, as well as the mechanisms of action.

## Methods

### Study location

**Fig 1:**
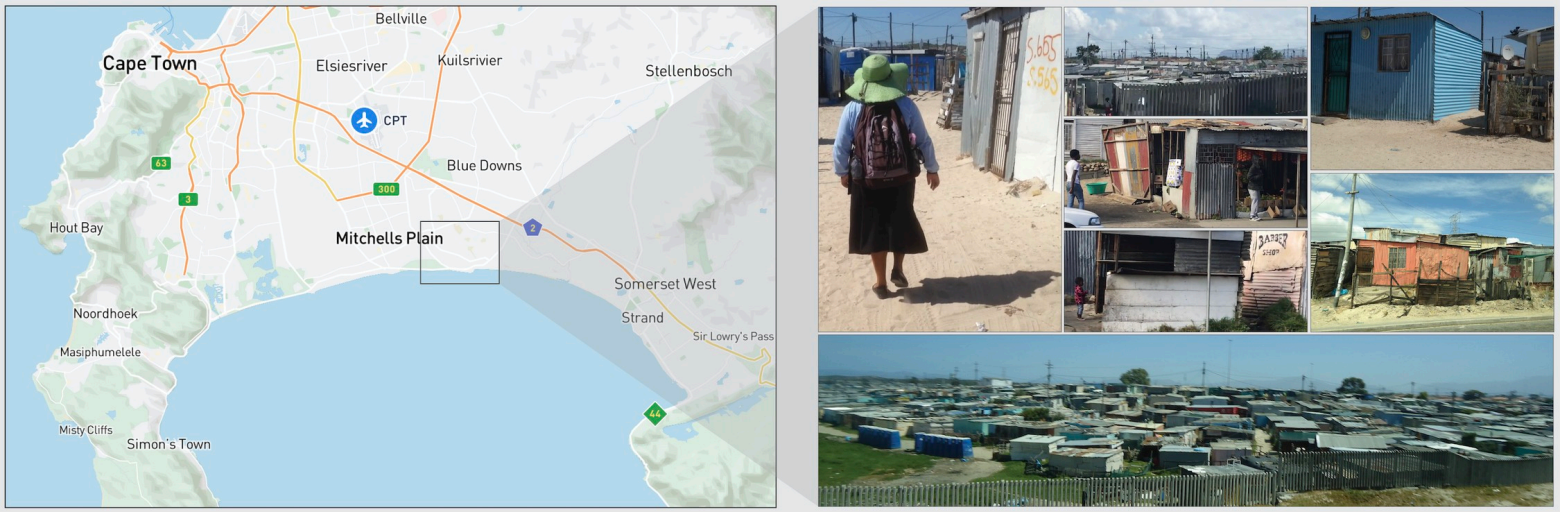
Philani MOVIE study setting. Left panel: map of the Western Cape Province, South Africa, (illustration by Sufian Ahmed). Right panel A: a Philani mentor mother walking between home visits. Right panel B: collage of homes and shops in Khayelitsha, South Africa. Right panel C: Bird’s eye view of Khayelitsha, a sprawing informal settlement (photos by Maya Adam). MOVIE, MObile Video Intervention for Exclusive breastfeeding.

This study was conducted in an underresourced region of the Western Cape Province [13,35]. This part of the province is characterized by high infant mortality rates and a low prevalence of EBF compared with country-wide statistics [36]. Within this region, Philani mentor mothers serve more than 100 neighborhoods [11], providing in-home health promotion counseling services and social support. Settlements in this region are characterized as either “formal” (containing government-constructed permanent dwellings made of brick and mortar), “informal” (containing community-constructed shacks made of wood, corrugated iron, and other available materials), or “mixed” (containing both types of dwellings). Mentor mothers are positive role models as well as frontline healthcare providers in their communities. Each mentor mother is trained in performing growth monitoring, counseling pregnant women and mothers on perinatal health, infant feeding, HIV and tuberculosis prevention and management, basic nutrition, early child development, and alcohol/drug avoidance [11,12,37]. Fig 1 shows the study setting.

### Trial design

We designed a stratified, cluster-randomized controlled trial, with mentor mothers as the unit of randomization [36]. Mentor mothers recruited pregnant women as participants in this trial during the enrollment period. Analyses of existing program data from Philani suggested that the mentor mother’s settlement type (formal, informal, or mixed) was the only significant predictor of EBF, among a range of covariates. We thus chose to stratify our randomization by settlement type, thereby attempting to balance the video intervention and control groups.

Based on prior research [9,38–45] we also expected several covariates to influence our primary outcome measure: (participant’s number of previous children, participant’s age, running water in the home, electricity in the home, participant’s employment status, and participant’s education level). We measured these covariates at baseline and adjusted for them in sensitivity analyses to increase the statistical efficiency of our estimation.

### Changes to trial design

At the outset of the trial, each mentor mother was living and counseling participants within her neighborhood. In October 2019, 5 months before data collection was terminated prematurely due to the Coronavirus Disease 2019 (COVID-19) pandemic, the provincial government altered Philani’s service demarcation. Roughly half of the mentor mothers at Philani were affected by the change and instructed to cease counseling the mothers who lived outside of their new demarcations. In our study, 12 mentor mothers (6 in the video intervention arm and 6 in the control arm) needed to be replaced by other mentor mothers, some of whom lived in settlement types that were different from those of the originally assigned mentor mother. The new demarcations led to a slight change in the distribution of settlement types in which the mentor mothers lived at endline (Intervention:Control = Formal 3:5, Informal 14:9, Mixed 25:27); however, the participants originally recruited remained in the study. For those participants whose mentor mother was replaced, data were collected by the replacement mentor mother. The COVID-19 pandemic and the changes in demarcation resulted in an unanticipated number of participants who were lost to follow-up, especially at the 5-month data collection point.

### Study size

Our sample size calculation was based on the primary outcomes: EBF at ages 1 and 5 months. We used standard methods for cluster-randomized controlled trials (with stratification, as well as with and without baseline covariate adjustment) to calculate our sample size [46]. The mentor mothers served as our unit of randomization. We assumed an intracluster correlation for each of our 2 primary outcomes of 0.1. Our power calculation was informed by routine program data describing the performance of the mentor mothers in our study and other sources of data on breastfeeding in South Africa [3–5,8,43,47,48]. Based on these data, we assumed that 40% of mothers exclusively breastfeed their infants at age 1 month and 10% of mothers exclusively breastfeed their infants at age 5 months. We further assumed that each mentor mother would enroll an average of 12 pregnant participants over the course of our trial. For the sample size calculation with baseline covariate adjustment, we assumed a correlation between the baseline measurements and the primary outcome of 0.30. We estimated that the trial would have 80% power to detect, at the 5% significance level, a 13-percentage point increase in the primary outcome at age 1 month and 9-percentage point increase in the primary outcome at age 5 months. These minimal detectable differences satisfied our condition for policy relevance (an improvement of more than 15 percentage points). As a result, we initially set the total sample size for outcome assessment at 840 pregnant women plus 20% (to allow for loss to follow-up), i.e., 1,008 pregnant women [36]. With the approval of our data safety and monitoring board (DSMB), we allowed enrollment to continue to 1,502 pregnant women to offset unanticipated data missingness that occurred over the December 2018 holiday period.

### Randomization

Faculty based at Heidelberg University in Germany performed the stratified randomization of the 84 mentor mothers, eligible for participation, using a computer-generated random allocation sequence. Randomization was stratified by settlement type. Email was used to transfer the allocations to Philani, where they were implemented by senior Philani staff overseeing the mentor mothers. We chose cluster randomization over individual randomization in this trial due to the organically occurring clusters formed by each mentor mother counseling the pregnant women within her neighborhood. This organization of mentor mothers’ work made individual participant randomization logistically challenging, while cluster randomization was both easier and aligned with community practice. Mentor mothers enrolled individual participants on a rolling basis, including checking eligibility criteria and eliciting informed consent.

### Participants

**Fig 2:**
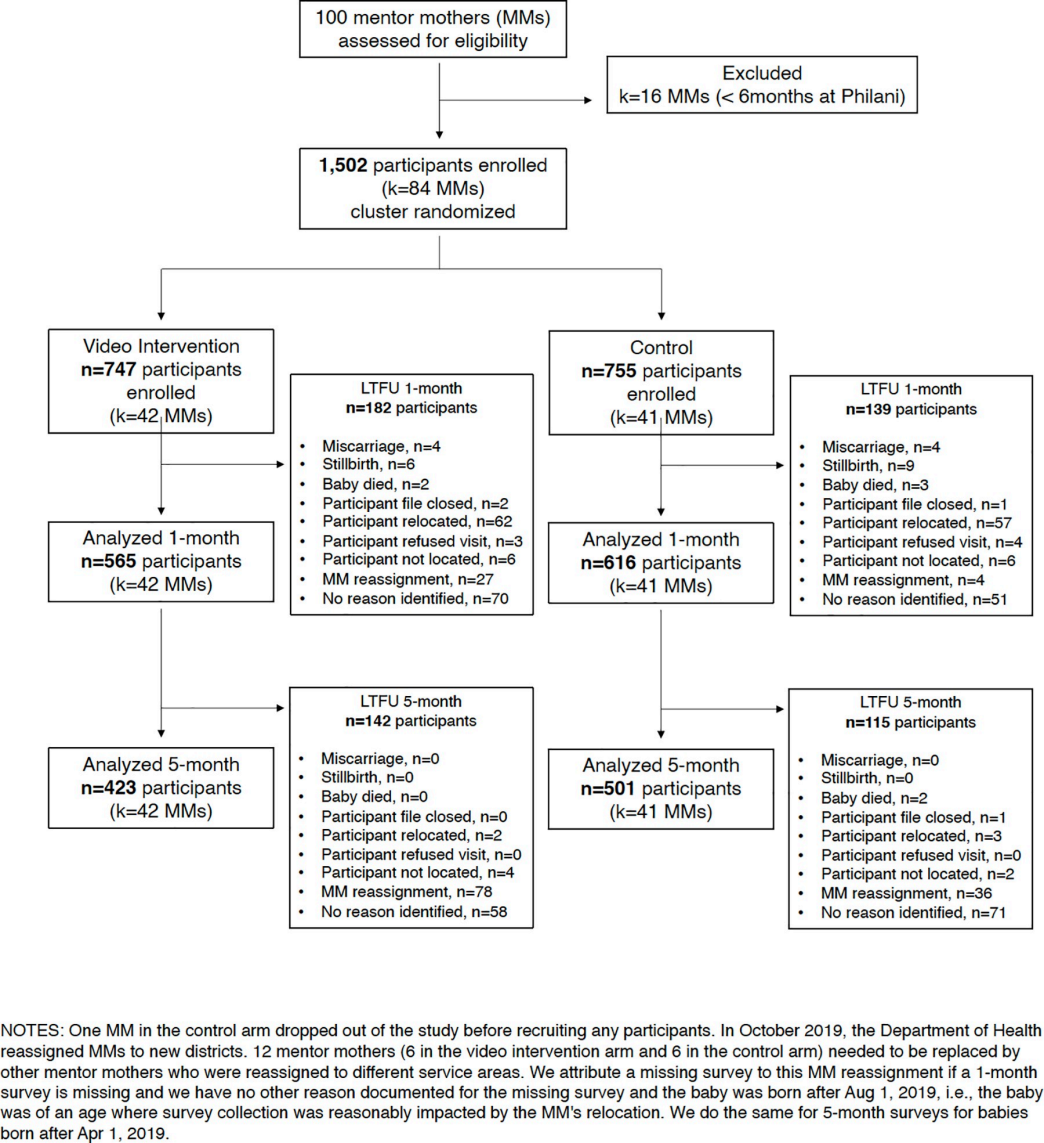
Participant flow diagram for the Philani MOVIE study. LTFU, loss to follow-up; MM, mentor mother; MOVIE, MObile Video Intervention for Exclusive breastfeeding.

A total of 1,502 women (age 18+ years) participated in our trial. Eighty-four mentor mothers recruited the participants. (Out of 100 mentor mothers working for Philani in the Western Cape at the beginning of this study, 16 were not eligible for study participation because they had been employed by Philani for less than 6 months.) Written, informed consent was collected from all participants by their mentor mother, prior to data collection. Participants were advised that they could exit the trial at any time. We originally intended that both forms of data collection, telephone and face-to-face, would be concluded when the last child of the enrolled participants reached age 5 months. However, we had to terminate face-to-face data collection 3 months prematurely due to the COVID-19 pandemic. Our DSMB allowed us to continue the telephone survey for outcomes data collection until the originally scheduled time point. Eligible pregnant participants were recruited between the 20th and 35th weeks of pregnancy. Fig 2 shows the participant flow diagram for this study.

### Intervention development and delivery

**Fig 3:**
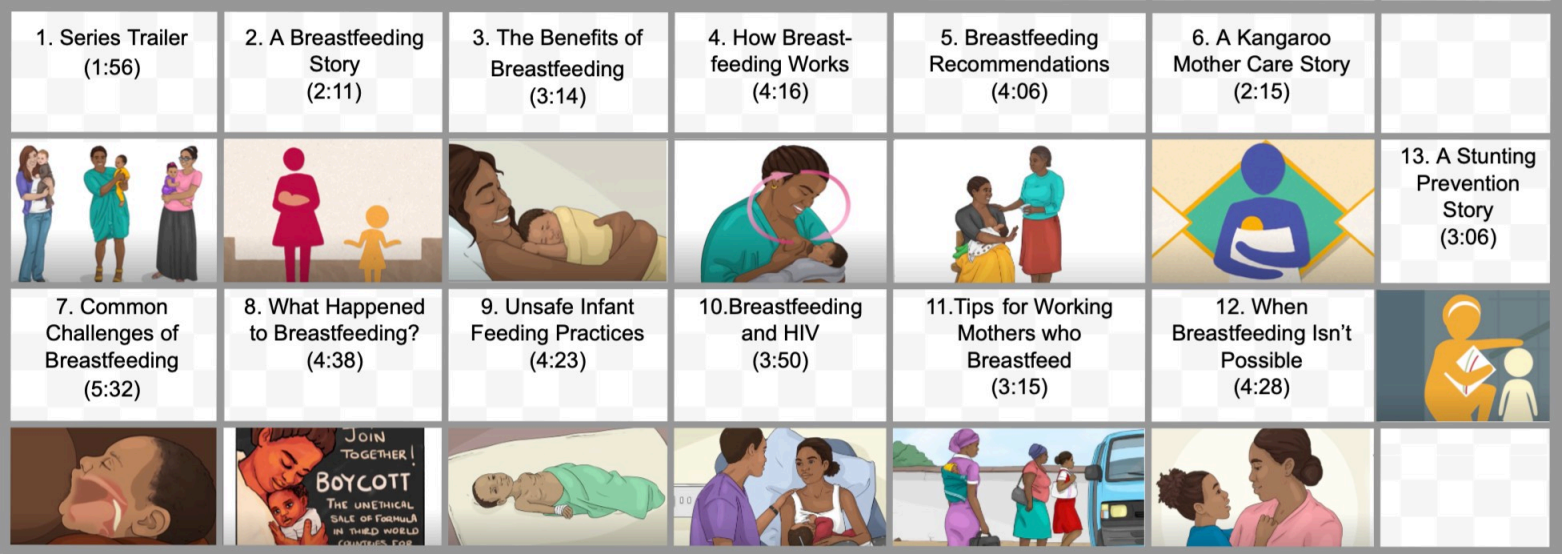
Philani MOVIE video topics and duration. Links to the videos used in the intervention can be found in the supporting information section (**S1 File)** at the end of this manuscript. MOVIE, MObile Video Intervention for Exclusive breastfeeding.

A total of 13 short (2 to 5 minutes) teaching videos comprised the Philani MOVIE intervention. We developed this content over a 10-month period, in collaboration with local government health advisors, Philani, and other local maternal–child health advisors. We used a human-centered design approach to tailor the intervention to address many of the specific needs and challenges that were identified by our target community. [36] The videos present learning objectives that are aligned with World Health Organization (WHO) recommendations for infant feeding. Videos were narrated in English and isiXhosa, the languages most commonly spoken among study participants. The primary health and motivational messages were illustrated by a local South African artist and interspersed with narratives from 3 South African celebrities and 4 community mothers. The videos avoided medical jargon, using simple language to convey each health message [49]. **Fig 3** provides an overview of the videos, including the titles, durations, and illustrative thumbnails showing one scene from each video.

In the intervention arm, mentor mothers delivered the Philani MOVIE videos. The videos were modular rather than sequential, allowing each mentor mother to tailor the order, frequency, and combination of videos used, to meet the individual needs of each pregnant woman and/or mother they supported. All video sequencing decisions were made by the mentor mothers. Philani ensures that the mentor mothers are trained to align their home-based health counseling with the individual needs and circumstances of the mothers they counsel. The intervention mentor mothers were asked to administer each video at least once per participating woman during the trial period. The tablets containing the videos were equipped to track video views. The video intervention mentor mothers were tasked with delivering the video intervention during their regular perinatal home visits, which typically include counseling on infant feeding methods.

### Trial outcome measures

Our outcome measures were based upon the WHO indicators for the study of infant feeding practices [50] and the most recent available, country-wide infant feeding data for South Africa [4]. All outcome measures pertain to the individual participant.

Our **primary outcomes** were short-term EBF (at 1 month) and long-term EBF (at 5 months), measured using both point-in-time (24-hour recall) and life-long (since birth) data. Prior research has recommended a dual approach to measuring EBF [51]. Primary outcomes data were collected via both face-to-face surveys and independent telephone surveys, as registered at clinicaltrials.gov (#NCT03688217). Where data points were missing from telephone surveys, these were replaced with data points from face-to-face surveys.

EBF at 1 month is widely used in the literature as an indicator of breastfeeding status [52,53]. We chose 5 months over 6 months as our second data collection point because we anticipated that real-world challenges in our study setting might result in some home visits occurring slightly after the desired date collection time point. Since complementary feeding is recommended from 6 months onward, we feared that the wording of our outcomes survey could yield misleading data from mothers whose babies had recently turned 6 months at the time of data collection and were appropriately receiving complementary foods.

Our **secondary outcomes** included the following:

1. Early initiation of breastfeeding (<1 hour after delivery, measured by recall on face-to-face and telephone surveys at 1 month);
2. Any breastfeeding at 1 and 5 months (based on 24-hour recall on face-to-face and telephone surveys);
3. Bottle-feeding (based on 24-hour recall on face-to-face and telephone surveys surveys);
4. Early introduction of complementary foods at 1 and 5 months (based on 24-hour and since-birth recall, measured by face-to-face and telephone surveys); and
5. Maternal knowledge at 1 and 5 months (measured via telephone surveys only).

**Table 1** summarizes our primary and secondary outcomes for this trial.

**Table 1:** Primary and secondary outcomes of the Philani MOVIE study.

Outcome	Definition	Measurement method
A. **PRIMARY OUTCOMES**		
1. Short-term EBF (24-hour recall)	Infant, age 1 month, was exclusively breastfed in the past 24 hours.	We measured these outcomes using both point-in-time (24-hour recall) and life-long (since birth) data. Prior research has recommended a dual approach to measuring EBF [51]. Primary outcomes data were collected via face-to-face and telephone surveys. The telephone surveys were conducted by an independent, telephone survey research company.
2. Long-term EBF (24-hour recall)	Infant, age 5 months, was exclusively breastfed in the past 24 hours.	
B. **SECONDARY OUTCOMES**		
1. Short-term EBF (since birth recall)	Infant, age 1 month, has been exclusively breastfed since birth.	
2. Long-term EBF (since birth recall)	Infant, age 5 months, has been exclusively breastfed since birth.	
3. Early initiation of breastfeeding	Infant was breastfed within the first hour of life (based on recall at 1 month).	We measured this outcome at the 1-month data collection point by face-to-face and telephone surveys.
4. *Any* breastfeeding at 1 month	Infant, age 1 month, received *any* breastmilk in the past 24 hours, even if not exclusively breastfed.	We measured these outcomes by face-to-face and telephone surveys at 1 month and 5 months, based on the most recent WHO indicators for infant feeding practices [50] and using questions adapted from the South Africa DHS 2016 [4].
5. *Any* breastfeeding at 5 months	Infant, age 5 months, received *any* breastmilk in the past 24 hours, even if not exclusively breastfed.	
6. Bottle-feeding	Infant, under age 6 months, was fed using a bottle with a nipple in the past 24 hours.	
7. Early introduction of complementary foods at 1 month (24-hour recall)	Infant, age 1 month, has received complementary foods in the past 24 hours.	We measured these outcomes by face-to-face and telephone surveys at 1 and 5 months. Participants responded to an infant feeding questionnaire adapted from previously published infant feeding measurement tools [54–56] and informed by the WHO indicators for infant feeding practices [50].
8. Early introduction of complementary foods at 5 months (24-hour recall)	Infant, age 5 months, has received complementary foods in the past 24 hours.	
9. Early introduction of complementary foods at 1 month (since birth recall)	Infant, age 1 month, has received complementary foods at some point since birth.	
10. Early introduction of complementary foods at 5 months (since birth recall)	Infant, age 5 months, has received complementary foods at some point since birth.	
11. Maternal knowledge at 1 month postdelivery	Maternal knowledge of breastfeeding current recommendations and basic health principles relevant to infant feeding measured at 1 month postdelivery.	We measured these outcomes by telephone survey only, using a 15-item, true–false questionnaire on maternal knowledge about infant feeding (adapted by the study team, from previously published breastfeeding knowledge assessment tools [45,57,58].
12. Maternal knowledge at 5 months postdelivery	Maternal knowledge of breastfeeding current recommendations and basic health principles relevant to infant feeding measured at 5 months postdelivery.	

DHS, Demographic and Health Survey; EBF, exclusive breastfeeding; MOVIE, MObile Video Intervention for Exclusive breastfeeding; WHO, World Health Organization.

### Blinding

To ensure that throughout the study period the 2 study arms did not receive differential treatment other than the randomly assigned intervention, all investigators and field team staff were blinded to the impact of intervention assignment on study outcomes, apart from one Stanford-based investigator (JJ). This investigator monitored preliminary intervention impact to report any concerns to the DSMB and to check on data collection integrity between the 2 study arms.

### Primary data analysis

The primary analysis was based on intention to treat (ITT) at the level of the individual participant. We used Poisson regression and adjusted standard errors for clustering at the level of the mentor mother. We chose modified Poisson models, because they generate estimates of risk ratios. This approach avoids the interpretational difficulties often associated with odds ratios and converges more easily than alternative approaches, such as negative binomial models, which also generate risk ratios [59]. We used generalized linear models with Gaussian distribution and identity link function for the continuous secondary outcomes (11 and 12—see **Table 1**). Single variable analyses were carried out for all prespecified and registered outcomes.

### Sensitivity analyses

In the first set of sensitivity analyses, we adjusted our estimates for the following 6 baseline covariates: participant’s number of previous children, participant’s age, running water in the home, electricity in the home, participant’s employment status, and participant’s education level. In the second set of sensitivity analyses, we used multiple imputation to account for missing data. Multiple imputation is commonly used to account for differential loss to follow-up by drawing from a distribution of likely values to replace missing data while adequately accounting for the uncertainty associated with such replacement. In multiple imputation, multiple datasets are created, then analyzed and combined to yield final results [60]. Mentor mother data, telephone surveys, and baseline variables were included in the multiple imputation model to generate a full dataset. We computed the number of multiple imputations required for this sensitivity analysis using the “howmanyimputations” package in Stata [61].

### Performance evaluation

At the conclusion of the trial, we conducted a mixed-methods performance evaluation including quantitative measurements of time usage and in-depth interviews with a subset of 26 mentor mothers (15 from the video intervention group and 11 from the control group) to gain a more nuanced interpretation of the trial results [62]. We asked mentor mothers about their personal experiences integrating tablets into their home visits as well as how they used the video intervention with participants. Maximum-variation purposive sampling was used to select the interview mentor mothers [63], and the interviews were continued until saturation and redundancy were reached [64,65]. Cape Town–based investigator, NJ, who gathered informed consent and conducted the interviews, speaks isiXhosa fluently and is trained in qualitative methods. We conducted weekly debriefings between the investigator conducting the interviews and 2 additional members of the research team, including the study lead [66]. These debriefings allowed the research team to glean early insights into the qualitative data. They also served to support and enhance the interview process for the research associate conducting the interviews, thereby enhancing the overall quality of the data [66]. All interviews were recorded, transcribed, translated into English, and quality controlled for consistency and accuracy. A qualitative analysis of the interview data has been completed using a grounded theory approach. We summarize key findings in this manuscript and report further details in an upcoming publication focused on the qualitative performance evaluation that accompanied this trial.

### Data collection and pretrial training

All participating mentor mothers were trained in obtaining written, informed consent, recording baseline variables, and collecting data about participants’ infant feeding practices using their tablets. The mentor mothers in the intervention arm were trained in accessing and showing the video interventions contained within the Digital Medic App. The app could be used offline while collecting usage data on frequency of video views that could then be downloaded upon subsequent connection to the internet. Infant feeding data, collected through the face-to-face surveys, was similarly stored, then downloaded when tablets were reconnected to the internet at Philani. After reconnection, all survey data were automatically transferred to the local research team for cleaning and analysis. Data were collected after the 1 month and 5 months postdelivery time points, first through face-to-face surveys conducted by the mentor mothers, with the majority of surveys collected within 4 weeks after the 1-month and 5-month birthdates. The surveys were translated into isiXhosa, and audio recorded versions of each question were available for mothers who preferred listening to the questions rather than reading them. Mentor mothers recorded survey responses on the tablets using the software, Survey CTO. All 84 mentor mothers carried tablets with the infant feeding surveys throughout the study period. Only tablets provided to the mentor mothers in the intervention arm were preloaded with videos. All mentor mothers received training on the use and care of their tablets, which were Android 8 devices with 16 GB of storage.

The face-to-face surveys, completed by participants on tablets, were used to triangulate data points collected through 30-minute telephone surveys, conducted after the 1-month and 5-month mentor mother visits. These telephone surveys were conducted by Social Surveys, a professional telephone research firm in South Africa. The face-to-face surveys were used to impute missing telephone survey data, and both data collection modalities were compared in a subset of participants to verify the data collected. We used only the telephone surveys to administer the knowledge assessment, asking participants to respond to 15 true/false questions related to the learning objectives in the video series. Finally, telephone surveys were used to detect potential contamination (i.e., to confirm directly, in a subset of participants, that the videos had been seen by participants enrolled in the video intervention arm and not seen by participants enrolled in the control arm). Computer-assisted telephone interviewing was used to enhance the accuracy of telephone survey data collection and reporting. All participants’ data were deidentified by local research staff in South Africa and stored in password-protected online storage drives.

### Theory of change

**Fig 4:**
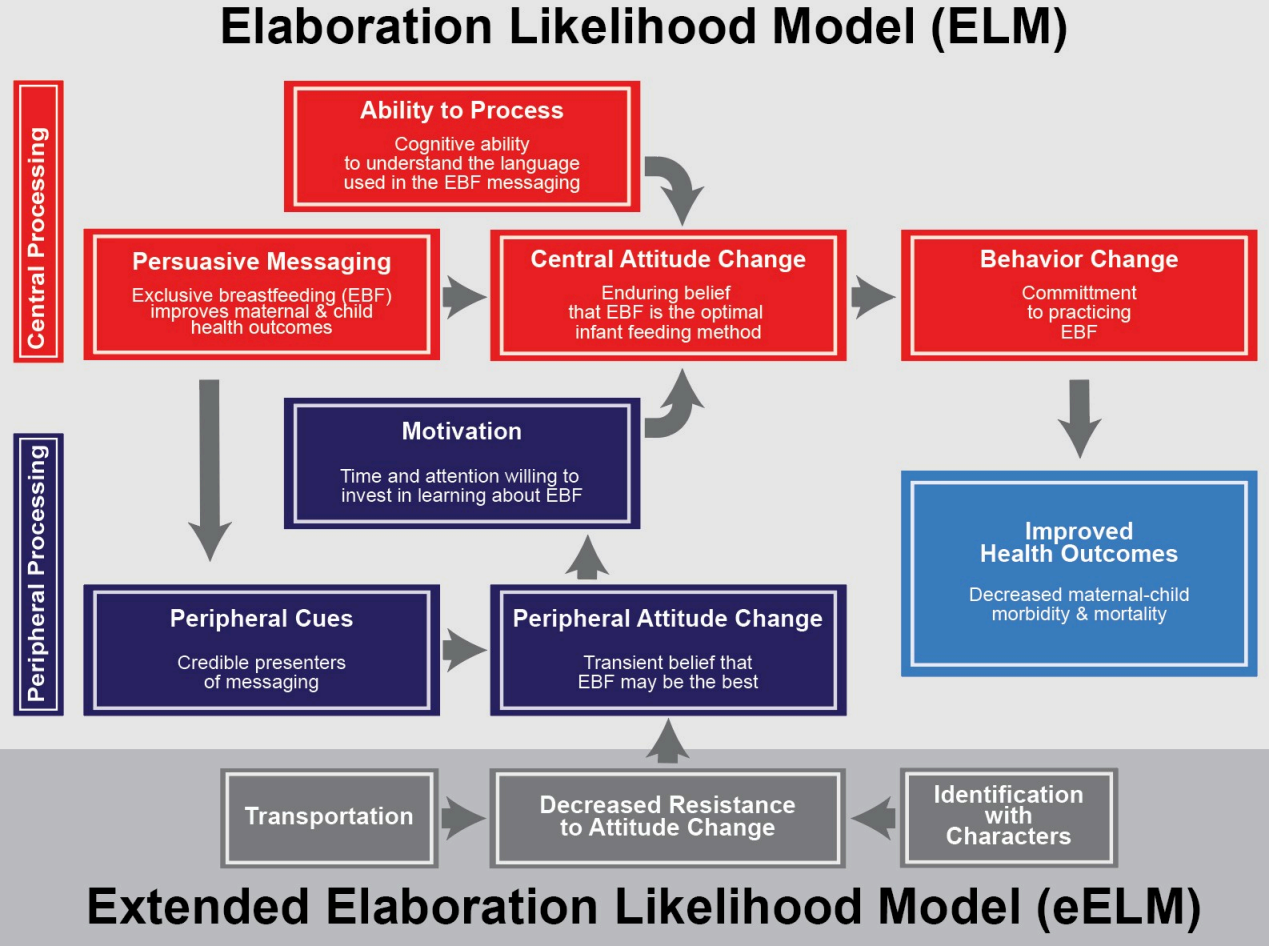
Theory of change. EBF, exclusive breastfeeding; eELM, extended ELM; ELM, Elaboration Likelihood Model.

The theoretical underpinnings of the elaboration likelihood model (ELM) [67] served as a foundation for the intervention tested in this study. The ELM describes 2 main pathways leading to attitude shifts that predict a desired behavioral outcome, like EBF. The first “central route” relies on an individual’s intrinsic motivation and cognitive decision-making, activated by the successful delivery of information. The second “peripheral route” relies on an enhancing transient motivation, influenced by peripheral cues that elicit emotion or identification within the learner. Peripheral cues can elicit temporary attitudinal shifts that support an individual’s intrinsic motivation and likelihood of processing informational messages via the central route [67]. Prior studies have leaned upon ELM, as well as the related extended elaboration likelihood model (eELM), to explain the impact of E–E on health-related attitudes and behaviors [18,21,68]. Fig 4 illustrates the intersection of the ELM and the eELM theoretical models with desired long-term health outcomes.

### Study governance and oversight

This study was overseen by a DSMB, consisting of a senior health systems researcher at the Norwegian Institute of Public Health and the Medical Research Council of South Africa, a senior biostatistician at the South African Medical Research Council, and a professor of pediatrics and health policy at Stanford University. All members of the DSMB have extensive expertise in health outcomes research. The DSMB met every 6 months throughout the duration of the study. The members of the DSMB helped us to evaluate the study progress, oversaw the study conduct, and were notified when face-to-face data collection needed to be terminated prematurely due to the COVID-19 pandemic.

### Ethics approval and consent to participate

Ethical approval was granted by the Stanford University IRB (Protocol #46667), the University of Stellenbosch IRB (Project ID #6318 HREC/UREC Reference #: N18/02/013), and the Ethics Committee of the Medical Faculty of Heidelberg University (Project #S-706/2018). All 3 committees are recognized Ethical Review Committees. Throughout the study, the investigators respected the principles of ethical research on human participants. Informed consent was obtained from all eligible participants in writing, before data collection began.

### Protocol

The protocol [36] for this study was published in April 2019.

## Results

Between November 2018 to December 2019, 1,502 participants were enrolled into the study by 83 mentor mothers, with 1 mentor mother (assigned to the control group) dropping out of the study prior to enrolling any participants. (See participant flow diagram in **Fig 2**). Mentor mothers enrolled, on average, just over 18 participants. The mean cluster size was 18.41 participants, with a standard deviation of 6.67 participants. We observed no significant difference in cluster size between study arms. In total, by study arm, 755 participants were enrolled by mentor mothers allocated to the control arm, and 747 participants were enrolled by mentor mothers assigned to the video intervention arm. Baseline characteristics of mentor mothers and participants in the 2 study arms were similar (see **Table 2**).

**Table 2:** Baseline descriptive characteristics by randomization arm.

	Video intervention		Control	
Characteristics	number (%)	*N*	number (%)	*N*
**Panel A: Mentor mothers**				
Age, years (median, range)	41 (29 to 59)	42	43.5 (30 to 58)	42
Years worked at Philani (median, range)	4 (1 to 14)	42	4.5 (2 to 13)	42
Settlement type				
Formal	5 (11.9%)	42	5 (11.9%)	42
Informal	10 (23.8%)	42	10 (23.8%)	42
Mixed	27 (64.3%)	42	27 (64.3%)	42
**Panel B: Participants**				
Age, years (median, range)	26 (18 to 46)	745	26 (18 to 48)	755
Number of previous children (median, range)	1 (0 to 6)	747	1 (0 to 6)	754
Employed outside of home	152 (20.4%)	744	122 (16.2%)	754
Infrastructure in home				
Electricity in the home	636 (85.1%)	747	690 (91.4%)	755
Running water in the home	476 (63.7%)	747	500 (66.4%)	753
Highest education				
Less than Secondary Education	36 (4.8%)	747	62 (8.2%)	755
Some Secondary Education	416 (55.7%)	747	434 (57.5%)	755
Completed Secondary Education or more	295 (39.5%)	747	259 (34.3%)	755
Baby’s gestational age, days (median, range)	205 (62 to 280)	743	204 (39 to 280)	750

Data given as number (percent) unless otherwise indicated.

At 1 month, a total of 321 participants (21.4%) were lost to follow-up (i.e., completing neither a face-to-face nor a telephone survey), with 139 participants (18.4%) lost from the control arm and 182 participants (24.4%) lost from the video intervention arm. At 5 months, a total of 578 participants (38.5%) were lost to follow-up, with 254 participants (33.6%) lost from the control arm and 324 participants (43.5%) lost from the video intervention arm. **Fig 2** illustrates the flow of the trial.

### Primary outcomes

#### One-month EBF

**Fig 5:**
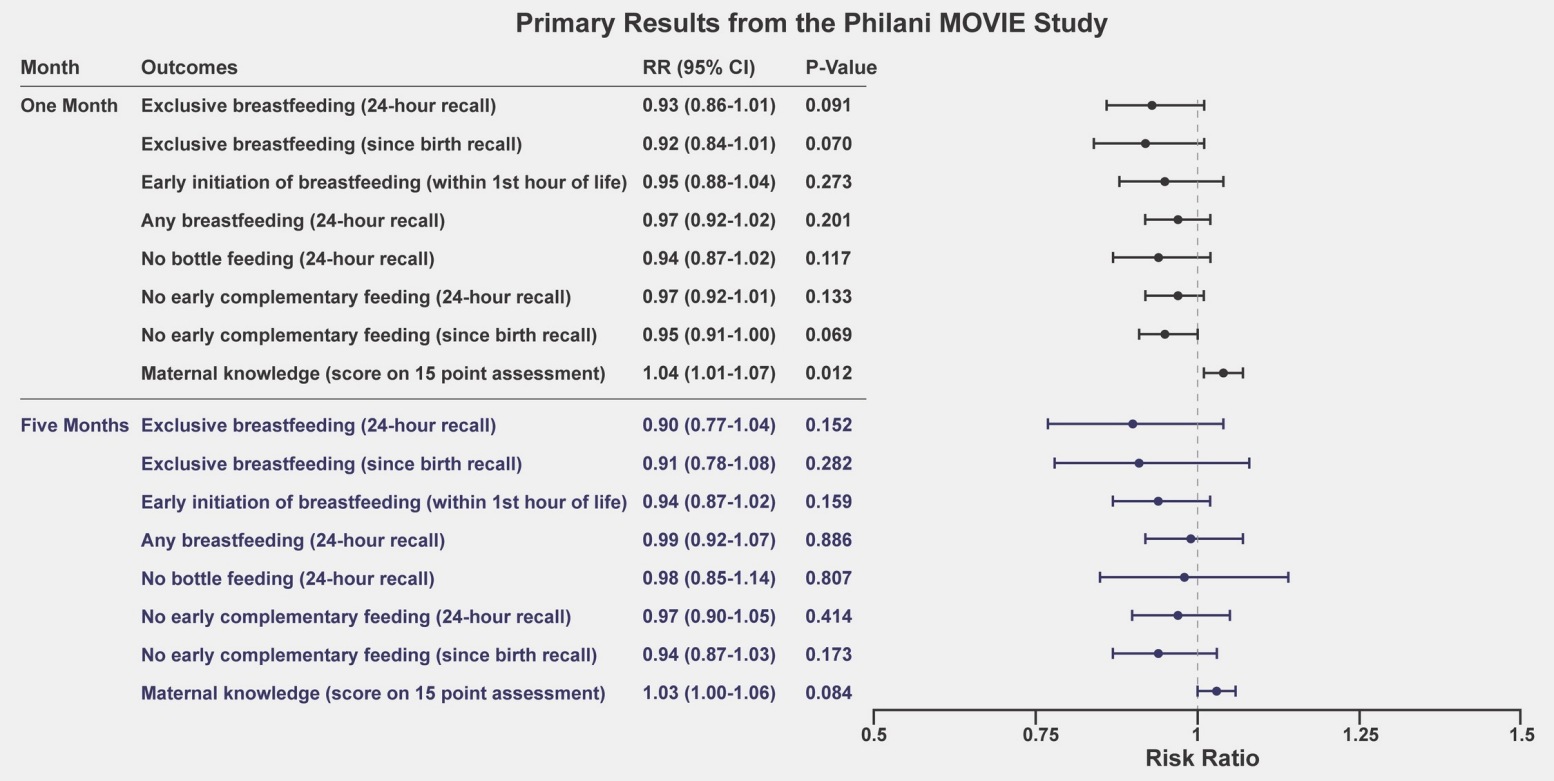
Infant feeding results at 1 month and 5 months. MOVIE, MObile Video Intervention for Exclusive breastfeeding; RR, risk ratio.

Overall, 71.5% of all participants reported exclusively breastfeeding in the last 24 hours at the 1-month follow-up, and 67.1% of all participants reported exclusively breastfeeding since birth (**Table 3**). We observed no statistically significant differences between the 2 study arms at 1 month for either of the primary outcomes: EBF in the last 24 hours (RR 0.93, 95% CI 0.86 to 1.01, *P* = 0.091) or since birth (RR 0.92, 95% CI 0.84 to 1.01, *P* = 0.070; **Fig 5**). The observed intracluster correlation coefficients for the 1-month primary outcomes were 0.05 (95% CI 0.01 to 0.09) for EBF in the last 24 hours and 0.05 (95% CI 0.01 to 0.09) for EBF since birth.

**Table 3:** EBF and other infant feeding practices at 1 and 5 months.

	Video intervention		Control	
Outcomes	number (%)	*N*	number (%)	*N*
**Panel A. One month**				
*Primary*				
EBF (24-hour recall)	389 (68.8%)	565	455 (73.9%)	616
EBF (since birth recall)	367 (65.0%)	565	435 (70.6%)	616
*Secondary*				
Early initiation of breastfeeding	363 (70.8%)	513	426 (74.2%)	574
Any breastfeeding (24-hour recall)	487 (86.2%)	565	549 (89.1%)	616
No bottle feeding (24-hour recall)	427 (75.6%)	565	494 (80.2%)	616
No early complementary feeding (24-hour recall)	479 (84.9%)	564	541 (88%)	615
No early complementary feeding (since birth recall)	467 (82.8%)	564	535 (86.9%)	616
Maternal knowledge score (mean, SD)	12.41 (2.00)	362	11.92 (2.11)	379
**Panel B. Five months**				
*Primary*				
EBF (24-hour recall)	216 (51.1%)	423	285 (56.9%)	501
EBF (since birth recall)	200 (47.3%)	423	259 (51.7%)	501
*Secondary*				
Early initiation of breastfeeding	276 (68.8%)	401	338 (72.8%)	464
Any breastfeeding (24-hour recall)	335 (79.2%)	423	399 (79.6%)	501
No bottle feeding (24-hour recall)	257 (60.8%)	423	310 (61.9%)	501
No early complementary feeding (24-hour recall)	235 (79.9%)	294	298 (82.5%)	361
No early complementary feeding (since birth recall)	226 (76.9%)	294	294 (81.4%)	361
Maternal knowledge score (mean, SD)	12.38 (2.03)	323	12.05 (2.11)	371

EBF, exclusive breastfeeding; SD, standard deviation.

**Fig 5** shows the primary outcomes of our study.

One-month results were robust to the inclusion of participant baseline covariates (24-hour recall, RR 0.99, 95% CI 0.92 to 1.07, *P* = 0.797; since birth, RR 0.99, 95% CI 0.91 to 1.07, *P* = 0.764; **S1 Table**) and consistent with analyses of data collected via independent telephone surveys only, with and without inclusion of baseline covariates (see **S2 and S3 Tables**).

#### Five-month EBF

Overall, 54.2% of all participants reported EBF in the last 24 hours at the 5-month follow-up, and 49.7% of all participants reported exclusively breastfeeding since birth (**Table 3**). We observed no statistically significant differences between the 2 study arms at 5 months for either of the primary outcomes: EBF in the last 24 hours (RR 0.90, 95% CI 0.77 to 1.04, *P* = 0.152) or since birth (RR 0.78, 95% CI 0.78 to 1.08, *P* = 0.282; **Fig 5**). The observed intracluster correlation coefficients for the 5-month primary outcomes were 0.05 (95% CI 0.15 to 0.36) for EBF in the last 24 hours and 0.05 (95% CI 0.15 to 0.35) since birth.

Five-month results were robust to the inclusion of participant baseline covariates (24-hour recall, RR 0.80, 95% CI 0.60 to 1.05, *P* = 0.109; since birth, RR 0.77, 95% CI 0.58 to 1.04, *P* = 0.087; see **S1 Table**) and consistent with analyses of data collected via independent telephone surveys only, with and without inclusion of baseline covariates (see **S2 and S3 Tables**).

**Table 3** shows EBF and other infant feeding practices, by study arm, at 1 and 5 months.

Our second set of sensitivity analyses, conducted to account for potential biases introduced by differential loss to follow-up, were similarly robust. Multiple imputation, both with and without baseline covariates, showed no significant change in our measured effects. **Table 4** shows results after multiple imputation, excluding and including baseline covariates.

**Table 4:** Results after multiple imputation, excluding and including baseline covariates.

Outcome	IRR (95% CI)	*P* value
**Panel A: No baseline covariates**		
One month		
EBF (24-hour recall)	1.01 (0.98 to 1.05)	0.588
No bottle feeding (24-hour recall)	1.00 (0.92 to 1.09)	0.996
Early initiation of breastfeeding	0.96 (0.89 to 1.04)	0.305
Maternal knowledge (score on 15 point assessment)	1.03 (0.99 to 1.06)	0.158
Five months		
EBF (24-hour recall)	0.84 (0.67 to 1.04)	0.114
EBF (since birth recall)	0.86 (0.69 to 1.07)	0.182
Maternal knowledge (score on 15 point assessment)	1.02 (0.98 to 1.06)	0.313
**Panel B: Including baseline covariates**		
One month		
EBF (24-hour recall)	0.97 (0.90 to 1.05)	0.441
No bottle feeding (24-hour recall)	1.01 (0.92 to 1.09)	0.896
Early initiation of breastfeeding	0.97 (0.90 to 1.05)	0.441
Maternal knowledge (score on 15 point assessment)	1.02 (0.98 to 1.05)	0.341
Five months		
EBF (24-hour recall)	0.82 (0.67 to 1.02)	0.074
EBF (since birth recall)	0.84 (0.68 to 1.05)	0.128
Maternal knowledge (score on 15 point assessment)	1.01 (0.97 to 1.05)	0.598

This table shows effects sizes from imputed outcomes and predictors using multiple imputation.

CI, confidence interval; EBF, exclusive breastfeeding; incidence risk ratio.

### Other infant feeding practices

**Table 3** shows the rates of other infant feeding practices measured at the 1-month and 5-month follow-ups. Overall, we observed that early initiation of breastfeeding was high, with 85% of participants reporting in the 1-month follow-up that they gave their baby breastmilk within the first hour of life. At the 1-month follow-up, 92% of mothers reported that their baby had received some breastmilk in the last 24 hours, and at the 5-month follow-up, 84% of mothers reported giving some breastmilk to their babies in the last 24 hours. At 1 month and 5 months, respectively, 81% and 63% of mothers reported no bottle feeding in the last 24 hours. Nearly all mothers reported no early complementary feeding at 1 month (95% for 24-hour recall and 94% for since-birth recall), and nearly three-quarters reported no early feeding at 5 months (73% for 24-hour recall and 72% for since-birth recall). We did not examine early complementary feeding at 5 months, because this measure was not applicable at this time point in an infant’s life. Across all additional infant feeding practices, we did not observe any statistically significant differences between the 2 study arms (**Fig 5**). Results were robust to inclusion of baseline covariates and multiple imputations and to an examination of outcomes using the independent telephone surveys (**S1–S3 Tables and Table 4**).

### Maternal knowledge

On average, mothers answered 12 questions correctly on the 15-point knowledge assessment administered via telephone at the 1- and 5-month time points. At 1 month, we observed a modest, but significant increase in maternal knowledge within the video intervention group (1.04, 95% CI 1.01 to 1.07, *P* = 0.012; **Fig 5**). This finding was robust to inclusion of baseline covariates (1.03, 95% CI 1.00 to 1.07, *P* = 0.026; **S1 Table**). We did not observe any difference in maternal knowledge between video intervention and control groups at the 5-month follow-up.

### Time tracking and video usage data

**Fig 6:**
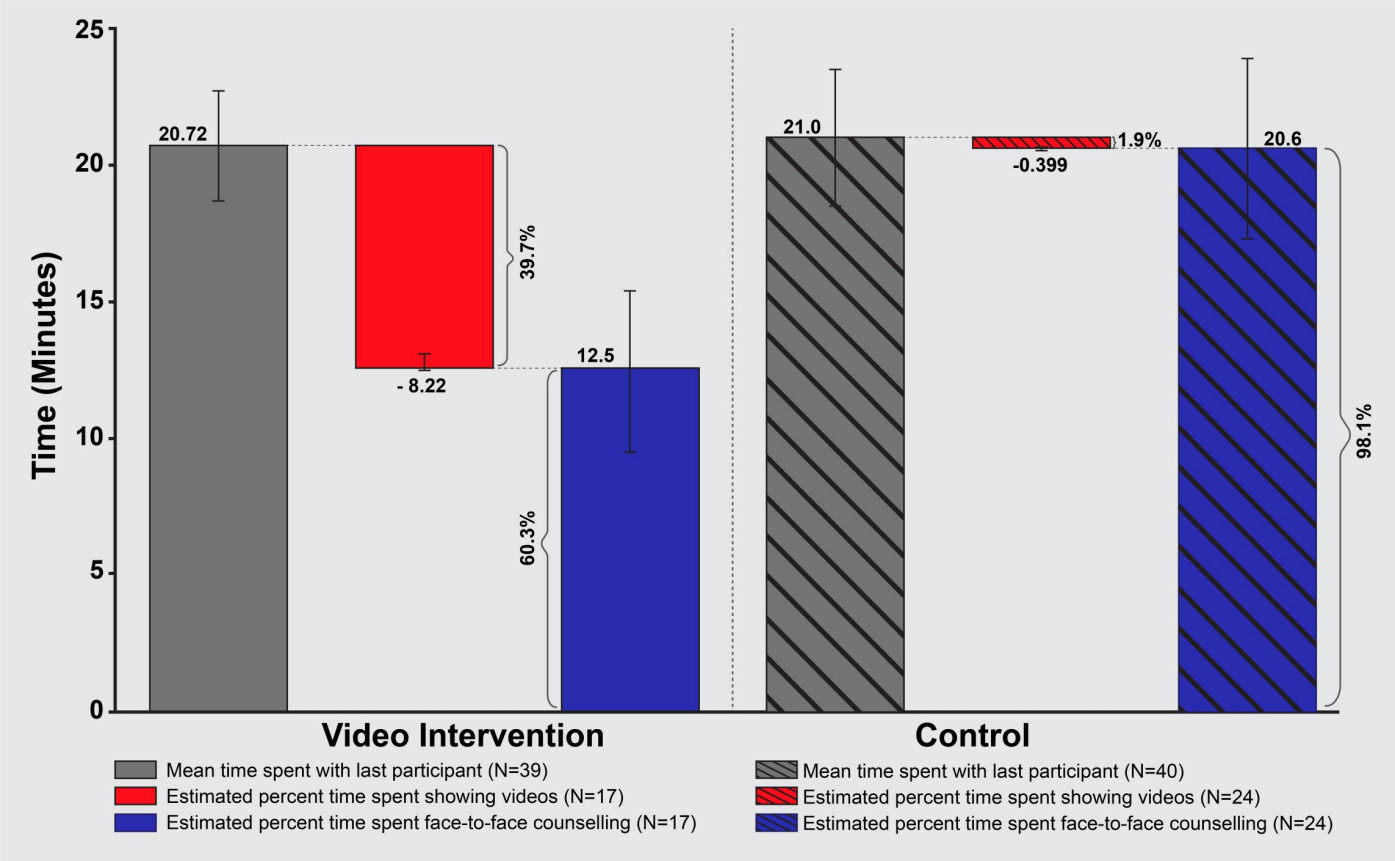
Per-visit time tracking and video viewing by arm. This figure illustrates the total mean time mentor mothers spent counseling participants as well as the mean proportion of that time spent watching videos versus face-to-face counseling in both groups.

During the course of the study, a subset of mentor mothers in the video intervention group (*N =* 39) and the control group (*N* = 40) reported on “time spent with participant during the last visit.” The mean estimated time spent with the last participant did not differ significantly between the 2 groups (Video Intervention = 20.72 minutes, Control = 21.03 minutes). Data on typical number of videos shown during each visit were collected from a further subset of video intervention (*N* = 17) and control (*N* = 24) mentor mothers. These data were used to estimate the mean percentage of visit time spent watching videos (Video Intervention arm = 39.7%, Control arm = 1.9%). These data are shown in **Fig 6** and presented in **Table 5**.

**Table 5:** Time usage during home visits.

	Video intervention				Control			
			95% CI				95% CI	
	*N*	mean	lower	upper	*N*	Mean	lower	upper
Number of minutes spent with last participant	39	20.72	18.65	22.78	40	21.03	18.50	23.55
% time showing videos	17	39.7	33.0	46.4	24	1.9	0.0	5.9
% time face to face couseling	17	60.3	53.6	67.0	24	98.1	94.1	100.0

Proportion of time showing videos was calculated using mentor mother reports of typical number of videos shown per session multiplied by the average video length. The number of minutes spent with the last participant was collected separately during unnanounced time checks with mentor mothers in January 2020.

CI, confidence interval.

### Performance evaluation

In-depth interviews with a subset of 26 mentor mothers (15 from the video intervention group and 11 from the control group) yielded several insights that helped us to contextualize and explain the equal outcomes observed in the 2 study arms. The vast majority of mentor mothers in the video intervention group valued the videos and used them frequently with the mothers they counselled. Tablet video tracking data showed that a total of 6,435 instances of the intervention videos were loaded by the mentor mothers across the duration of the trial. Mentor mothers described the videos as supportive of their health promotion efforts in 3 main ways:

1. By reducing the burden on the mentor mothers to perform 100% of the health counseling verbally, the mentor mothers perceived a “freeing up of time.” The mentor mothers further reported that they used this time to perform other health-related tasks, such as charting or completing referrals, during the home visit. Quantitative measurements of time usage indicate that mentor mothers in the intervention group, on average, showed videos for approximately 40% of their home visit time.
2. The mentor mothers felt that the videos increased the consistency of messaging across participants and improved general interest in infant feeding. Many mentor mothers described the usefulness of the intervention for motivating participants to dedicate time and attention to the consistent messages delivered in the videos. Several mentor mothers reported that participants would spontaneously express interest in watching additional or new videos, upon arrival of the mentor mother.
3. The mentor mothers felt that the messages presented in the videos underscored and legitimized their advice in general. Because the videos echoed the early perinatal health messages mentor mothers had delivered prior to video viewing, they felt the mothers they counselled were more likely to subsequently trust them and value their expertise.

Across both groups, video intervention and control, the tablets themselves were perceived to support the work of the mentor mothers. Mentor mothers in both study arms reported that the tablets enhanced their perceived authority in the community, by signifying that they were employed by a well-funded, well-established program, and by allowing them to demonstrate technological skills in front of the mothers they counselled. Mentor mothers in both groups reported that the surveys themselves stimulated new discussions around infant feeding definitions, practices, and even benefits. We will report further details on these findings in an upcoming publication focused on the qualitative performance evaluation nested within this trial.

## Discussion

To our knowledge, this study is the first to measure the effect of a mobile, video health intervention, delivered by CHWs, on infant feeding practices in an underresourced setting. We observed no difference in effects on both our primary endpoint (EBF) and secondary endpoints in this randomized controlled trial. However, our nested performance evaluation showed that the videos replaced approximately two-fifths of direct human interaction between the mentor mothers and trial participants. Together, the trial-based causal impact evaluation and the performance evaluation results suggest that the video intervention was as effective for promoting healthy infant feeding practices as face-to-face counseling, with the same investment of time. In hindsight, the unintended replacement of face-to-face counseling time with video viewing is highly plausible because mentor mothers have a limited amount of time for home visits and the availability of the videos did not increase mentor mothers’ time budget per visit.

Several policy recommendations follow from our findings. Firstly, mobile video interventions could be effectively deployed for routine support of pregnant women and new mothers in vulnerable communities in South Africa. Ideally, these deployments should be in addition to the other care that pregnant women receive, rather than replacing any of that care. In communities that are becoming increasingly tech-savvy, CHWs are well positioned to facilitate access to video interventions, for example, by helping community mothers to download them onto their mobile devices and providing online links for later viewing on shared devices. Secondly, our results imply that E–E videos could form the basis of community health promotion campaigns on infant feeding and other behaviors essential for healthier living. Evidence-based video interventions should be made universally available, in particular to vulnerable communities, whether or not people also have access to CHWs.

Video-based, mHealth interventions should ideally be used as an addition—rather than as a replacement—for human engagement on infant feeding practices and other health behaviors. As smartphone ownership and internet access continue to rise in LMICs [28], widespread distribution of video health interventions becomes increasingly feasible. Where mentor mothers or other CHWs are scarce, video health interventions provide some of the functions that CHWs used to deliver, freeing up time activites that cannot be “task-shifted” to mobile devices, such as physical examinations and treatment support. At the same time, our qualitative findings suggest that CHWs may benefit from having a tablet or smartphone to support their crucial role in community health promotion. Through collaboration, academic institutions, community-based organizations, and government health authorities could create a new generation of mobile video health interventions, which supplement rather than replace CHW activities. The ongoing development and evaluation of these interventions could contribute to enhanced community-based health promotion, overcoming literacy barriers and increasing knowledge transfer to those living in underresourced settings. Such interventions could catalyze improvements in health decision-making and health outcomes for mothers and children around the world.

### Strengths and limitations

As the first trial to measure the effect of a CHW-delivered mobile, video health intervention on the infant feeding behaviors of a large population of participants living in an underresourced setting, this trial constitutes a valuable addition to the literature on mobile video health at the community level. Furthermore, the unintended downstream consequence of the intervention provide authentic insights well beyond our anticipated research aims.

As anticipated when implementing pragmatic trials in “real-world” settings, we experienced several unforeseen events that led to the setbacks described above and below. We were able to partially overcome these using data triangulation and additional sensitivity analyses during the data analysis phase. During our study, the provincial government mandated changes in Philani’s service demarcation, which led to the reassignment of some mentor mothers. This change, as well as the dropout of one mentor mother before she had recruited any participants, required 6 new mentor mothers in both the intervention and the control arm of this study. Because not all mentor mothers could be replaced by mentor mothers from the same settlement types, the distribution of the settlements where mentor mothers lived had become slightly imbalanced across intervention and control arms by the end of the study. However, in the cases where mentor mothers needed to be replaced, the originally recruited participants remained in the trial, within their original study arm, and their infant feeding data were collected by the replacement mentor mother. Since the baseline characteristics of the participants remained constant and balanced throughout the study, we would not expect that the unanticipated mentor mother replacements have biased our results. Similarly, the loss of a single mentor mother (1 out of 84) who never recruited any participants is unlikely to have introduced substantial bias.

The replacement of some mentor mothers combined with the outbreak of the COVID-19 pandemic contributed to a high loss to follow-up, especially at the 5-month data collection point. To address the potential for biases associated with differential loss to follow-up, we reestimated our effect sizes following multiple imputation of missing data. The results from these additional analyses suggest that our original results are robust despite significant data missingness.

The fact that the videos were widely used despite these “real-world” challenges within the CHW programs highlights the programmatic robustness and promise of mobile E–E videos. While we could not firmly establish the effectiveness of the videos in the randomized controlled trial, the interpretation of the trial results in light of the insights from the the nested performance evaluation suggests that the intervention was likely as effective as CHW counseling. The performance evaluation also showed that the videos were successfully implemented, highly valued by mentor mothers, and well received by community mothers. Future research should explore the feasibility and desirability of direct-to-mother video intervention delivery as well as the differential effect of such delivery pathways compared with the CHW-mediated delivery approach.

## Conclusions

For underresourced communities, increasing access to effective video health interventions could support CHWs and their communities of care, by promoting life-saving behaviors, such as EBF. To this end, mobile video interventions could play an important role, supporting existing CHW programs through “task-shifting” of counseling activities and by enhancing the perceived authority of CHWs. For those living without access to CHWs, effective mobile video interventions could serve as a lifeline, connecting health services with the hardest to reach communities. As mobile technology becomes increasingly available, health services, academic institutions, and community and government health organizations have a responsibility to collaborate in creating, evaluating, and distributing effective mobile video interventions to support the most vulnerable individuals in our global community.

## Supporting information

S1 CONSORT Checklist A completed CONSORT checklist for this trial. (DOCX)

S1 Table Primary results including baseline covariates. (DOCX)

S2 Table Exclusive breastfeeding rates and infant feeding practices at 1 and 5 months (telephone survey data). (DOCX)

S3 Table Primary results excluding and including baseline covariates (telephone survey data). (DOCX)

S1 File Links to the videos used in the intervention. (DOCX)

## Abbreviations

CHW: community health worker
COVID-19: Coronavirus Disease 2019
DSMB: data safety and monitoring board
EBF: exclusive breastfeeding
E–E: entertainment–education
eELM: extended elaboration likelihood model
ELM: elaboration likelihood model
ITT: intention to treat
LMICs: low- and middle-income countries
mHealth: mobile health
MOVIE: MObile Video Intervention for Exclusive breastfeeding
WHO: World Health Organization
