# Supplementary material for: Evaluation of a community-based mobile video breastfeeding intervention in Khayelitsha, South Africa: The Philani MOVIE cluster-randomized controlled trial
Source: PLoS Med. 2021 Sep 28;18(9):e1003744. doi: 10.1371/journal.pmed.1003744 (PMC8478218; doi:10.1371/journal.pmed.1003744)
Supplement: S1 Table — (DOCX) [file pmed.1003744.s002.docx]

| **S1 Table. Primary results including baseline covariates** | | | | | |
| --- | --- | --- | --- | --- | --- |
|  | One month | |  | Five months | |
| Outcome | IRR (95% CI) | P-value |  | IRR (95% CI) | P-value |
| Primary |  |  |  |  |  |
| Exclusive breastfeeding (24-hour recall) | 0.99 (0.92 to 1.07) | 0.797 |  | 0.80 (0.60 to 1.05) | 0.109 |
| Exclusive breastfeeding (since birth recall) | 0.99 (0.91 to 1.07) | 0.764 |  | 0.77 (0.58 to 1.04) | 0.087 |
|  |  |  |  |  |  |
| Secondary |  |  |  |  |  |
| Early initiation of breastfeeding | 0.96 (0.87 to 1.06) | 0.419 |  | 0.93 (0.81 to 1.07) | 0.311 |
| Any breastfeeding (24-hour recall) | 0.98 (0.93 to 1.02) | 0.257 |  | 0.93 (0.84 to 1.03) | 0.173 |
| No bottle feeding (24-hour recall) | 1.01 (0.92 to 1.09) | 0.896 |  | 0.86 (0.66 to 1.13) | 0.281 |
| No early complementary feeding (24-hour recall) | 1.01 (0.98 to 1.05) | 0.526 |  | 0.82 (0.67 to 1.02) | 0.074 |
| No early complementary feeding (since birth recall) | 1.01 (0.98 to 1.05) | 0.448 |  | 0.84 (0.68 to 1.05) | 0.128 |
| Maternal knowledge (score on 15 point assessment) | 1.03 (1.00 to 1.07) | 0.026 |  | 1.02 (0.99 to 1.05) | 0.148 |
|  |  |  |  |  |  |
| NOTES: This table shows outcomes collected through surveys administered by mentor mothers via tablets at the one-month and five-month follow-up points. Maternal knowledge measures were collected separately via independent telephone survey. For the early complementary feeding measures, we exclude surveys administered when babies were older than 6 months. Baseline covariates include running water in the home, electricity in the home, number of previous children, participant age, participant employed outside the home, highest education completed. | | | | | |
