# Supplementary material for: Evaluation of a community-based mobile video breastfeeding intervention in Khayelitsha, South Africa: The Philani MOVIE cluster-randomized controlled trial
Source: PLoS Med. 2021 Sep 28;18(9):e1003744. doi: 10.1371/journal.pmed.1003744 (PMC8478218; doi:10.1371/journal.pmed.1003744)
Supplement: S2 Table — (DOCX) [file pmed.1003744.s003.docx]

| **S2 Table. Exclusive breastfeeding (EBF) and infant feeding practices at 1 and 5 months: telephone surveys only** | | | | | |
| --- | --- | --- | --- | --- | --- |
|  | One month | |  | Five months | |
| Outcome | number (%) | N |  | number (%) | N |
| Primary |  |  |  |  |  |
| Exclusive breastfeeding (24-hour recall) | 515 (66.4%) | 776 |  | 367 (52.9%) | 694 |
| Exclusive breastfeeding (since birth recall) | 483 (62.2%) | 776 |  | 337 (48.6%) | 694 |
|  |  |  |  |  |  |
| Secondary |  |  |  |  |  |
| Early initiation of breastfeeding | 431 (65.5%) | 658 |  | 358 (66.8%) | 536 |
| Any breastfeeding (24-hour recall) | 658 (84.8%) | 776 |  | 536 (77.2%) | 694 |
| No bottle feeding (24-hour recall) | 595 (76.7%) | 776 |  | 432 (62.2%) | 694 |
| No early complementary feeding (24-hour recall) | 626 (82.3%) | 761 |  | 408 (84.5%) | 483 |
| No early complementary feeding (since birth recall) | 618 (80.2%) | 771 |  | 396 (82.0%) | 483 |
| NOTES: Data given as number (percent) unless otherwise indicated. Data collected through independent telephone surveys. Early initiation of breastfeeding only asked of participants who reported giving babies any breastmilk at the time of the survey. For the early complementary feeding measures, we exclude surveys administered when babies were older than 6 months. | | | | | |
