# Supplementary material for: Evaluation of a community-based mobile video breastfeeding intervention in Khayelitsha, South Africa: The Philani MOVIE cluster-randomized controlled trial
Source: PLoS Med. 2021 Sep 28;18(9):e1003744. doi: 10.1371/journal.pmed.1003744 (PMC8478218; doi:10.1371/journal.pmed.1003744)
Supplement: S3 Table — (DOCX) [file pmed.1003744.s004.docx]

| **S3 Tables. Primary results excluding and including baseline covariates: telephone surveys** | | | | | | |
| --- | --- | --- | --- | --- | --- | --- |
|  | One month | |  | Five months | |  |
| Outcome | IRR (95% CI) | P-value |  | IRR (95% CI) | P-value |  |
| **Panel A: No baseline covariates** |  |  |  |  |  |  |
|  |  |  |  |  |  |  |
| Primary |  |  |  |  |  |  |
| EBF (24-hour recall) | 0.93 (0.84 to 1.04) | 0.218 |  | 0.95 (0.80 to 1.13) | 0.552 |  |
| EBF (since birth recall) | 0.92 (0.81 to 1.04) | 0.189 |  | 0.97 (0.82 to 1.15) | 0.91 |  |
|  |  |  |  |  |  |  |
| Secondary |  |  |  |  |  |  |
| Early initiation of breastfeeding | 0.97 (0.89 to 1.07) | 0.553 |  | 1.03 (0.91 to 1.16) | 0.682 |  |
| Any breastfeeding (24-hour recall) | 0.97 (0.91 to 1.04) | 0.384 |  | 1.03 (0.94 to 1.14) | 0.475 |  |
| No bottle feeding (24-hour recall) | 0.97 (0.88 to 1.07) | 0.505 |  | 1.04 (0.89 to 1.21) | 0.640 |  |
| No early complementary feeding (24-hour recall) | 0.96 (0.89 to 1.03) | 0.239 |  | 1.00 (0.93 to 1.09) | 0.906 |  |
| No early complementary feeding (since birth recall) | 0.94 (0.87 to 1.02) | 0.145 |  | 0.97 (0.89 to 1.06) | 0.486 |  |
|  |  |  |  |  |  |  |
| **Panel B: Including baseline covariates** |  |  |  |  |  |  |
|  |  |  |  |  |  |  |
| Primary |  |  |  |  |  |  |
| EBF (24-hour recall) | 0.93 (0.83 to 1.03) | 0.156 |  | 0.97 (0.82 to 1.15) | 0.729 |  |
| EBF (since birth recall) | 0.91 (0.81 to 1.03) | 0.140 |  | 1.01 (0.84 to 1.21) | 0.911 |  |
|  |  |  |  |  |  |  |
| Secondary |  |  |  |  |  |  |
| Early initiation of breastfeeding | 0.96 (0.88 to 1.05) | 0.392 |  | 1.02 (0.90 to 1.16) | 0.762 |  |
| Any breastfeeding (24-hour recall) | 0.98 (0.92 to 1.04) | 0.417 |  | 1.05 (0.95 to 1.15) | 0.348 |  |
| No bottle feeding (24-hour recall) | 0.97 (0.88 to 1.07) | 0.510 |  | 1.06 (0.91 to 1.23) | 0.455 |  |
| No early complementary feeding (24-hour recall) | 0.96 (0.89 to 1.02) | 0.198 |  | 1.00 (0.93 to 1.08) | 0.993 |  |
| No early complementary feeding (since birth recall) | 0.94 (0.87 to 1.02) | 0.128 |  | 0.97 (0.89 to 1.05) | 0.476 |  |
| NOTES: This table shows outcomes collected independent telephone surveys. For the early complementary feeding measures, we exclude surveys administered when babies were older than 6 months. Baseline covariates include running water in the home, electricity in the home, number of previous children, participant age, participant employed outside the home, highest education completed. | | | | | | |
