# Supplementary material for: Evaluation of a community-based mobile video breastfeeding intervention in Khayelitsha, South Africa: The Philani MOVIE cluster-randomized controlled trial
Source: PLoS Med. 2021 Sep 28;18(9):e1003744. doi: 10.1371/journal.pmed.1003744 (PMC8478218; doi:10.1371/journal.pmed.1003744)
Supplement: S1 File — (DOCX) [file pmed.1003744.s005.docx]

Stable links to intervention videos

The Philani MOVIE Study

The videos listed below were used by community health workers in South Africa, called ‘mentor mothers’, to promote health infant feeding, including exclusive breastfeeding, in our trial.

Videos can be accessed through the Digital Medic Mobile App, which can be downloaded on mobile Android and iOS devices:

<https://digitalmedic.stanford.edu/general/digital-medic-app-now-available-on-ios-and-android/>

Videos can also be viewed or downloaded at the links below:

1. [Series Trailer](https://drive.google.com/file/d/19IEQSA3UbqgsVRk3ZQYzeD_EvjYDfadi/view?usp=sharing)

2. [A Breastfeeding Story](https://drive.google.com/file/d/1ZPiVBfdNEXQVvcjsjLKoVG42SE8WWIAB/view?usp=sharing)

3. [The Benefits of Breastfeeding](https://drive.google.com/file/d/1vLcMOW4sVnuscUGug_Kq6mmR4oi3voNa/view?usp=sharing)

4. [How Breastfeeding Works](https://drive.google.com/file/d/16cAbYEwPhdEWoDz8uOt6P7wj3sqUu8My/view?usp=sharing)

5. [Breastfeeding Recommendations](https://drive.google.com/file/d/1mz9-7FNqOKRqQgHm_w-9X_kQzNTYc-N_/view?usp=sharing)

6. [A Kangaroo Mother Care Story](https://drive.google.com/file/d/1Fn6i8vEeWRGJxIKHxh5kLbQp1f_sFqRd/view?usp=sharing)

7. [Common Challenges of Breastfeeding](https://drive.google.com/file/d/1a3kHlSWmKbU7KHsB-wGTLSCXPPhjBAH5/view?usp=sharing)

8. [What Happened to Breastfeeding?](https://drive.google.com/file/d/1l4ltj_NVQUg0-5nvalPqogSKHHKyrGrz/view?usp=sharing)

9. [Unsafe Infant Feeding Practices](https://drive.google.com/file/d/1hC5RB-38_rSFHPzj130G_4jwofIJb-qK/view?usp=sharing)

10. [Breastfeeding and HIV](https://drive.google.com/file/d/1NRUwkQozdtffs01FjtcrZAfxS1OcCA4H/view?usp=sharing)

11. [Tips for Working Mothers who Breastfeed](https://drive.google.com/file/d/1Je7Y-Wue-u_IxJcW4Iv6BZeh9RAmG7b9/view?usp=sharing)

12. [When Breastfeeding Isn’t Possible](https://drive.google.com/file/d/1OyjYtyA_M8iEbLDyCs9gfBNidofrk2GH/view?usp=sharing)

13. [A Stunting Prevention Story](https://drive.google.com/file/d/1KWUdTROq96OVgekrXtGXOIdBzwmLgu24/view?usp=sharing)

Please contact Maya Adam MD ([madam@stanford.edu](mailto:madam@stanford.edu)) or [digitalmedic@stanford.edu](mailto:digitalmedic@stanford.edu) with any questions.
